# High Fracture Toughness of 1D Copper‐Based MOP Electrode Enables Fast‐Charging Lithium‐Ion Batteries

**DOI:** 10.1002/advs.75786

**Published:** 2026-05-22

**Authors:** Mingli Li, Zhenzhen Wu, Pan Yang, Di Zhao, Mengyang Dong, Muhammad Tayyab Ahsan, Lei Zhang, Shanqing Zhang, Yun Wang

**Affiliations:** ^1^ School of Environment and Science Gold Coast Campus Griffith University Southport Australia; ^2^ Institute for Sustainable Transformation School of Chemical Engineering and Light Industry Guangdong University of Technology Guangzhou China

**Keywords:** fast‐charging, fracture toughness, Li‐ion batteries, one‐dimensional metal–organic polymer, π‐d conjugation

## Abstract

Fast charging technology in lithium‐ion batteries (LIBs) is critically dependent on the mechanical robustness of electrode materials, which must withstand significant stress and strains during rapid cycling. However, a comprehensive study of the relationship between mechanical property and battery performance remains rare, particularly under fast‐charging conditions. In this work, we bridge this knowledge gap by developing a one‐dimensional conductive metal–organic polymer (MOP, i.e., Cu‐DDA) that exhibits high fracture toughness for fast charging. Moreover, the robust π‐d conjugation structure of Cu‐DDA minimizes lattice deformation and volume expansion, thereby preserving structural stability under high current densities. As a result, the Cu‐DDA cathode achieves an attractive reversible capacity of 190 mAh g^−1^ at a high current density of 15 A g^−1^ and remarkable capacity retention of 78% after 400 cycles at 5 A g^−1^. This study demonstrates the significant impact of inherent mechanical properties, providing important design insights for next‐generation fast‐charging electrode materials.

## Graphical abstract

Traditional electrode materials suffer from particle cracking and rapid capacity fade during fast charging, largely originating from their low fracture toughness. This work introduces a one‐dimensional Cu‐based metal–organic polymer, in which strong π‐d conjugation and *π–π* stacking endow the framework with high fracture toughness, simultaneously enabling high reversible capacity and stable cycling under fast‐charging conditions.

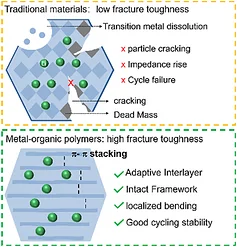

## 1 Introduction

The rapid domination of lithium‐ion batteries (LIBs) in a variety of applications, from consumer electronics to electric vehicles, has created a growing demand for fast charging technology. Achieving efficient fast charging is closely related to the mechanical properties of the electrode material [1, 2, 3]. During fast electrochemical cycling, the repetitive insertion/removal of lithium ions induces cumulative lattice strain, as well as volume expansion and contraction in the electrode material. These mechanical stresses can lead to particle fragmentation and accelerated capacity degradation after exceeding the fracture threshold of the material. Therefore, optimizing the mechanical properties of the material is essential to balance electrochemical performance and structural durability under extreme operating conditions.

**SCHEME 1:**
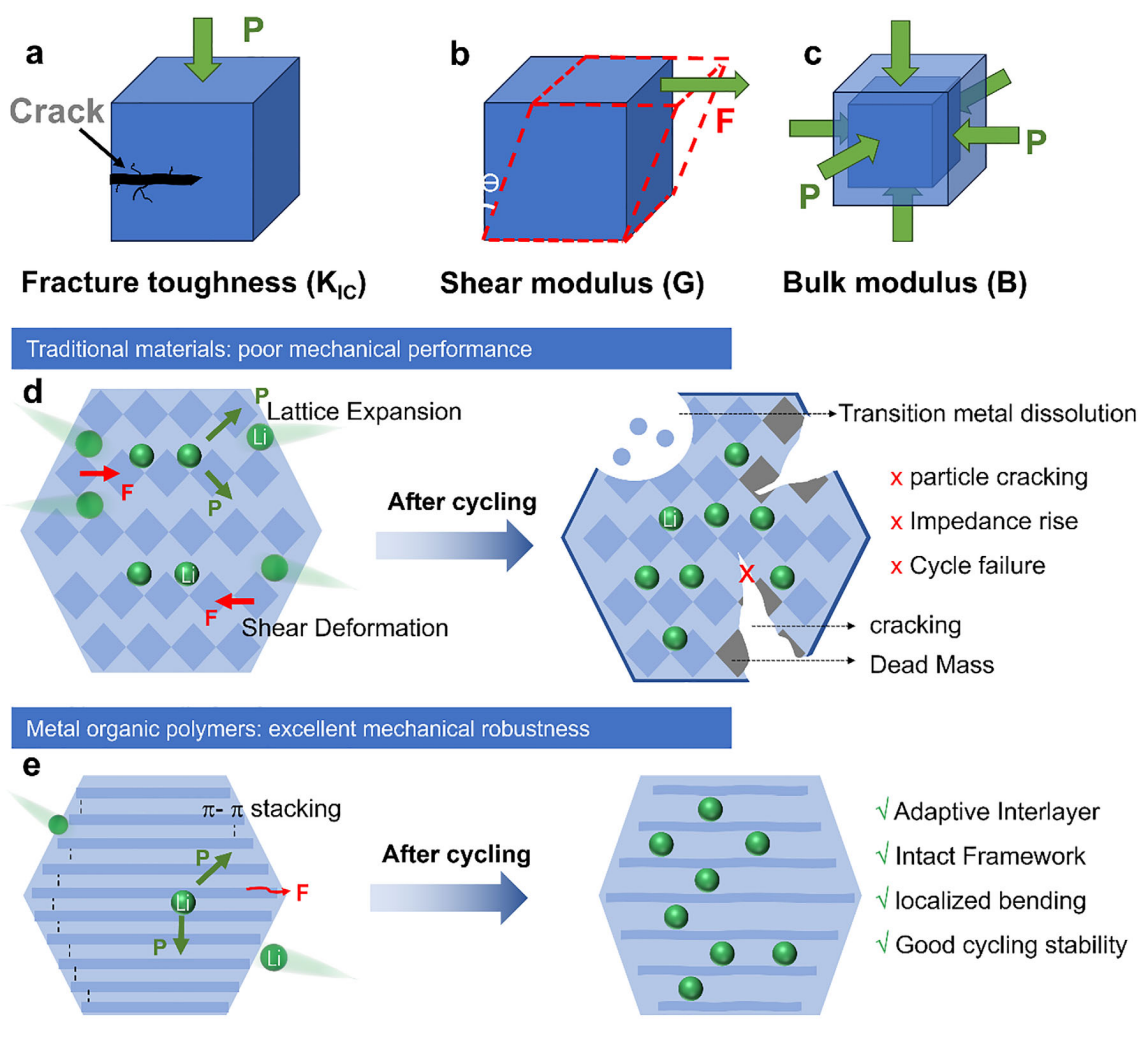
Significant mechanical parameters. (a) Fracture toughness K_IC_, (b) Shear modulus, and (c) Bulk modulus. Schematic comparison between (d) traditional materials and (e) metal–organic polymers. “P” and “F” represent the external pressure (P) and force (F).

Among mechanical descriptors, fracture toughness (K_IC_, Scheme 1a) is one of the most critical metrics, as it quantifies the resistance to crack propagation and structural failure [4]. For common covalent compounds and ionic compounds, the K_IC_ can be obtained through the following equation [5],

(1) $$K_{IC}={V_{0}}^{1/6}\;\times G\times {\left(B/G\right)}^{1/2}$$

where V_o_ is the volume per atom. G denotes the shear modulus (Scheme 1b). Materials with low G values tend to undergo shear sliding and cause cracking propagation. Moreover, B corresponds to the bulk modulus for reflecting the ability to resist the volume change (Scheme 1c) [6]. The B/G is devoted to the Pugh ratio. A B/G value greater than 1.75 indicates that the material exhibits ductile behavior [7]. Equation (1) suggests that the high fracture toughness can be engineered by optimizing the mechanical properties of materials.

In general, high fracture toughness demonstrates that the electrode material can withstand the stresses and strains induced by high current densities during charge/discharge cycling, reducing the risk of cracking and ensuring stability over long cycling times [5]. Conversely, low K_IC_ leads to the cyclic and anisotropic crystal deformation, e.g., *c*‐axis collapse, and irreversible intergranular or transgranular fracture rather than stress‐relieving deformation. Such structural disintegrations sever electronic pathways, create freshly exposed cathode surfaces that trigger recursive parasitic reactions, and eventually result in impedance rise and catastrophic cycling failure (Scheme 1d) [8]. For instance, the widely used commercial LiCoO_2_ cathode shows a low K_IC_ value of about 1.7 ± 0.4 MPa m^1/2^ [9], making it prone to cracking during cycling [10]. Besides, Liu et al. reveal that as the increasing content of Ni in layered Li(Ni*_x_* Mn*_y_* Co_z_)O_2_ (i.e., NMC, x, y, z are the contents of Ni, Co, and Mn, x + y + z = 1), the B/G values are similar, but the G decreases, leading to a lower K_IC_ of NMC with a high Ni content [11]. This result indicates that the high‐Ni NMC has a higher risk of cracking during electrochemical cycling despite its superior specific capacities.

Metal–organic polymers (MOPs) are regarded as promising candidates for next‐generation energy storage materials due to their tunable pore structure, high specific surface area, and abundant ion storage sites [12]. However, most MOPs present poor mechanical strength because of their highly porous structure, leading to low‐rate capability and short cycling lifetime [13]. This vulnerability significantly limits their practical application in fast‐charging and high‐power batteries [13]. Stimulated by the high K_IC_ value of 4.0 ± 0.6 MPa m^1/2^ on the low‐dimensional graphene structure [4], low‐dimensional conductive metal–organic polymers (c‐MOPs) with a similar structure have similar potential mechanical robustness and structural integrity as graphene. This is because *π–π* stacking and a planar structure of low‐dimensional c‐MOPs facilitate efficient stress dispersion, energy dissipation, and topological adaptability, which can effectively enhance the mechanical properties [14, 15].

Among them, one‐dimensional (1D) c‐MOPs not only retain key structural features, such as π‐conjugated backbones and coordinative metal–ligand frameworks, but also their linear topology can introduce distinctive anisotropic behavior [16]. The aligned molecular chains in 1D architecture can enable the framework to accommodate deformation through localized bending and elongation modes. Such flexibility could be advantageous under the repeated mechanical and chemical stresses experienced during rapid lithium insertion/extraction [15, 17, 18]. Despite these merits, the correlation between the electrochemical performance of 1D c‐MOPs and their K_IC_ remains poorly understood.

Herein, we used a 1D c‐MOP, assembled by the Cu^2+^ and 1,5‐Diamino‐4,8‐dihydroxy‐9,10‐anthraceneedione (DDA), as a model molecular system to investigate the influence of the mechanical properties on the battery performance of electrode materials. Cu‐DDA shows excellent mechanical properties, with a K_IC_ value as high as 2.5 MPa m^1/2^. Benefited by this merit, the Cu‐DDA cathode shows good rate performance at high current density (190 mAh g^−1^ at 15 A g^−1^), and great cycling cycles (95.6% retention after 100 cycles at 0.5 A g^−1^, and 78% retention at 5 A g^−1^ for 400 cycles). This work underscores the importance of fracture toughness as an indispensable parameter for the rational design of mechanically resilient and fast‐charging electrode materials.

## 2 Results and Discussion

**FIGURE 1:**
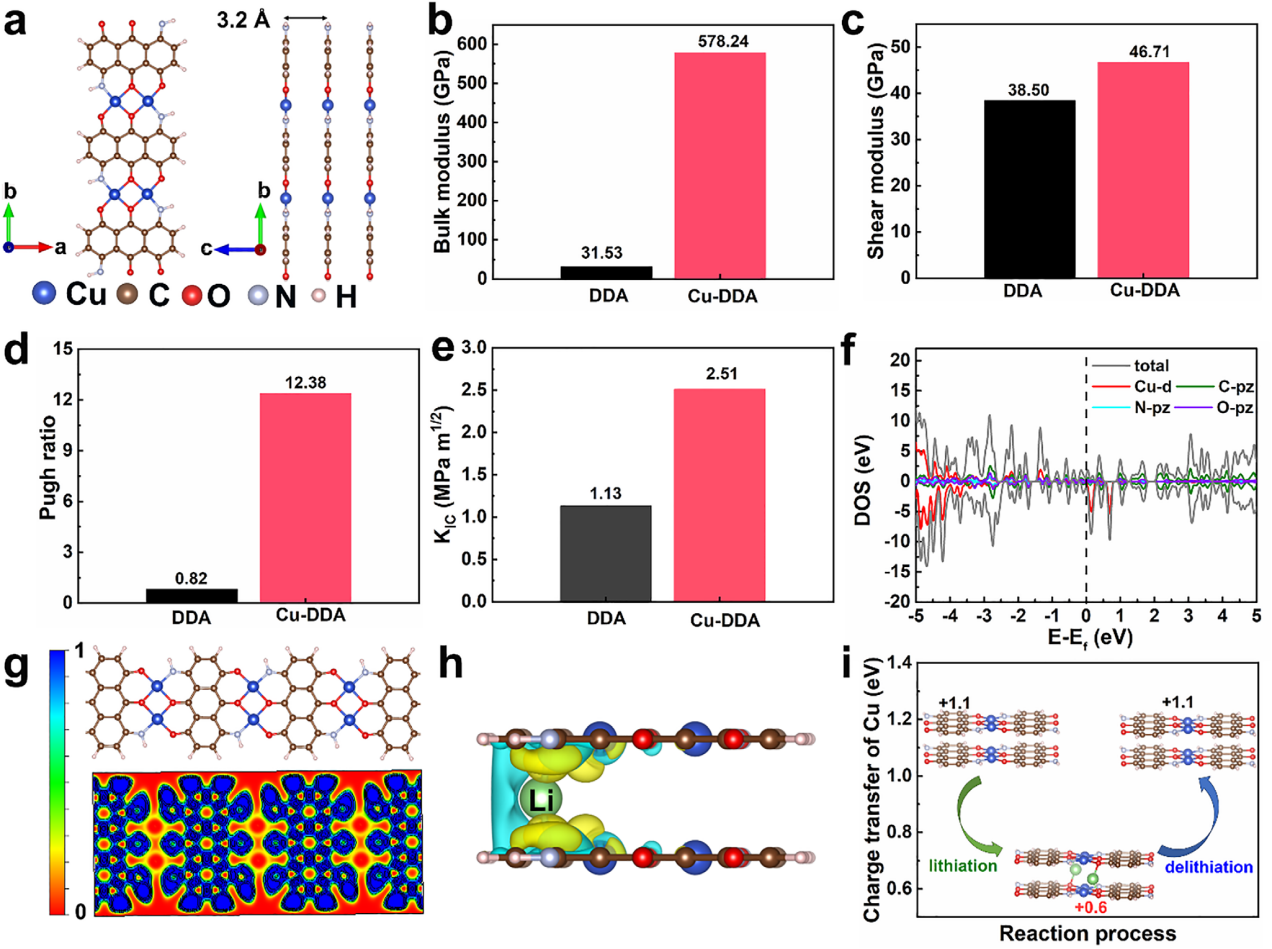
Structural and mechanical properties of Cu‐DDA MOPs. (a) One unit molecular structure for Cu‐DDA MOPs from top and side views, respectively. (b–e) Comparison of the critical mechanical parameters of DDA ligand and Cu‐DDA MOPs, including Bulk modulus (b), Shear modulus (c), Pugh ratio (d), and K_IC_ value (e). (f) DOS figure of Cu‐DDA MOPs. (g) ELF figure of Cu‐DDA MOPs. (h) Charge density difference between Cu‐DDA MOPs and the absorbed Li atom. The blue and yellow areas denote electron loss and gain, respectively. (i) The Bader transfer of Cu in the lithiation and delithiation state.

The Cu‐DDA MOP forms a nanorod structure through the self‐assembly of DDA ligands, each coordinating with two copper ions. They further stack along the *c*‐axis through π–π interactions, forming a layered architecture with an interlayer spacing of ∼3.2 Å (Figure 1a) [19, 20]. The DDA ligand is derived from an anthraquinone scaffold bearing coplanar hydroxyl, carbonyl, and amino groups, which facilitate coordination with metal centers and promote ordered self‐assembly. [21]. The three adjacent benzene rings on the anthraquinone create a large conjugated *𝜋*‐surfaces that underpins efficient intrachain electron delocalization and contributes to the overall mechanical robustness [22]. The mechanical properties of Cu‐DDA were computed based on the Voigt‐Reuss‐Hill approximation. Compared with the DDA ligand, the 1D Cu‐DDA MOP presents a higher B and similar G (Figure 1b,c). Moreover, the related Pugh ratio of Cu‐DDA indicates its priority of ductility (Figure 1d). Interestingly, Cu‐DDA presents excellent fracture toughness with a high K_IC_ value of 2.51 MPa m^1/2^ (Figure 1e), which surpasses that of many traditional electrode materials (Table S5).

The increased B, G, and K_IC_ values can be ascribed to the special structural and electronic properties of Cu‐DDA. As shown in the density of states (DOS) figures (Figure 1f), the d orbitals of the Cu atoms overlap with the p_z_ orbitals of the C, O, and N atoms in the ligands, facilitating effective electron delocalization across the framework. Furthermore, the electron localization function (ELF) result for Cu‐DDA further validates the significant electron delocalization at the Cu nodes and the high electron localization around the DDA ligand, which supports the stability of the whole framework and the fast electron transfer (Figure 1g) [23]. The strong π‐d conjugation induces spatial redistribution of delocalized electrons within the conjugation planes, while *π–π* stacking between the chains facilitates additional through‐space electron transfer. These two mechanisms act synergistically to enable rapid charge compensation at high current densities while maintaining structural stability. The combined effect of π‐d conjugation and *π–π* stacking optimizes the B and G values of the Cu‐DDA to enhance the K_IC_ [24].

Besides, the structural durability endowed by π‐d conjugation within the 1D MOP and *π–π* conjugation between MOPs ensures fully reversible charge transfer dynamics of Cu‐DDA for Li^+^ storage. The charge density difference reveals that after absorbing a Li ion, a significant charge transfer between Li and the Cu‐DDA MOP, implying the applicability of Cu‐DDA in Li storage (Figure 1h) [25, 26]. As shown in Figure 1i, the charge transfer of the Cu atom in Cu‐DDA decreases from + 1.1 to + 0.6 during the adsorption of Li^+^, and returns to + 1.1 after desorption. The ability of the Cu center to return to its initial state after delithiation demonstrates that the durable Cu‐DDA structure enables fully reversible charge‐transfer  [27].

In addition, the π‐d conjugation and the *π–π* conjugation also provide the Cu‐DDA MOP with good electrical conductivity, which was shown to be 0.089 S cm^−1^ by a four‐point probe test via the four‐point probe testing (Figure S1). This conductivity stems from its narrow bandgap (0.41 eV and low LUMO energy level (−3.03 eV), which synergistically enhances the electron mobility and electron trapping ability. (Figure S2) [28, 29, 30]. Besides, the LUMO orbitals are predominantly localized at Cu nodes and adjacent functional groups (Figure S3), suggesting that these sites can act as active centrers for Li^+^ storage while stabilizing the Li^+^ through charge transfer [31].

**FIGURE 2:**
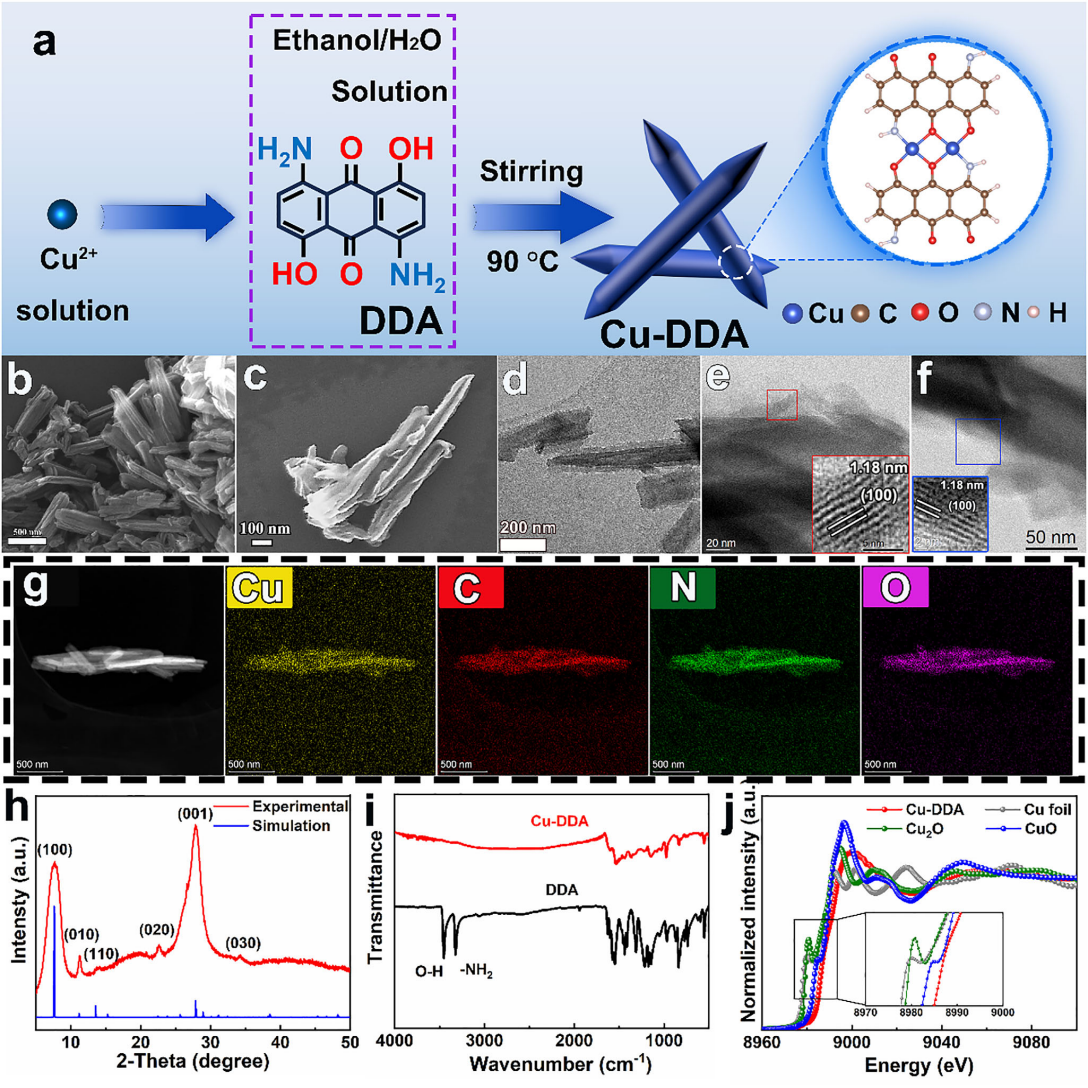
(a) Schematic illustration of the synthesis of Cu‐DDA MOP. (b–g) morphology and element identification of Cu‐DDA MOPs: low magnification SEM (b), high magnification SEM (c), TEM images (d), HRTEM images (e‐f), and elements mapping images (g). (h) XRD experimental and simulated model of Cu‐DDA. (i) FTIR of Cu‐DDA and DDA. (j) XANES spectrum of Cu‐DDA, Cu_2_O, CuO, and Cu foil.

Stimulated by the theoretically predicted high fracture toughness and promising Li^+^ storage performance of the 1D Cu‐DDA, its nanorod was prepared by the coordination between Cu(CH_3_COO)_2_ and DDA ligand (Figure 2a) [20]. The corresponding morphologies and microstructures of Cu‐DDA were investigated by scanning electron microscopy (SEM) and transmission electron microscopy (TEM). The SEM images demonstrate that the surface of the Cu‐DDA nanorod is smooth and clean (Figure 2b,c). The TEM image in Figure 2d further verifies the one‐dimensional nanorod structures. In the high‐resolution TEM (HRTEM) images shown in Figure 2e,f, clear lattice spacing fringes with an interplanar distance of 1.18 nm can be indexed to the (101) crystal planes of Cu‐DDA. In Figure 2g, the element mapping images confirm the presence of Cu, N, O, and C elements on the nanorod with uniform distribution.

Further spectroscopic characterizations were incorporated to identify the molecular information and fine structures in Cu‐DDA. Powder x‐ray diffraction (PXRD) analysis (Figure 2h) confirms the crystallinity of Cu‐DDA, where the experimental pattern is matched with the simulated one. The pattern of Cu‐DDA at 7.6°, 11.3°, 22.6°, and 27.9° can be indexed to the (110), (010), (020), and (001) planes, respectively, indicating the phase purity and structural integrity of the synthesized coordination framework. Besides, the distance between the two layers is approximately 3.2 Å. Such tight *π–π* stacking thereby suppresses the interlayer shear sliding [32]. The presence of π‐d conjugation and *π–π* stacking can also guarantee the high stability of Cu‐DDA in the reaction process^.^[33] Nitrogen adsorption/desorption analysis reveals a specific surface area of 31 m^2^ g^−1^ with a pore size of 5 nm, suggesting that most pores in the framework of Cu‐DDA are mesopores (Figure S5) [34]. The presence of mesopores can facilitate electrolyte penetration and shorten the ion diffusion pathway, thereby enabling rapid ion transport. According to the Fourier‐transform infrared (FT‐IR) spectra (Figure 2i), both the −NH_2_ peak at 3322 cm^−1^ and the ‐OH peak at 3454 cm^−1^ disappear in Cu‐DDA, implying that the N and O on DDA coordinate with Cu^2+^ and form the Cu‐DDA MOPs [20, 21, 35, 36].

As shown in Figure 2j, x‐ray absorption near‐edge structure (XANES) of Cu‐DDA exhibits a lower energy edge similar to that of CuO, implying the + 2 valence of Cu ion in Cu‐DDA [29]. The extended x‐ray absorption fine structure (EXAFS) spectrum shows the main peaks at 1.55 and 1.99 Å, which can be attributed to the Cu‐O and Cu‐N paths [20]. Moreover, the absence of long‐range Cu─Cu interactions further supports the isolated coordination environment within the Cu‐DDA framework (Figure S6) [37]. X‐ray photoelectron spectroscopy (XPS) further confirms the presence of Cu, N, and O in the Cu‐DDA (Figure S7a). The Cu 2*p* peaks reveal the copper ions in Cu‐DDA are bivalent (Figure S7b) [38, 39], while the O 1s peak shifted toward higher binding energy [40], and the N 1s peak exhibited a new signal corresponding to the Cu─N bond (Figure S7c,d) [35]. Solid‐state electron paramagnetic resonance (EPR) spectroscopy presents a strong signal at g = 2.13, which is the characteristic value of the single unpaired electron in the 3d^9^ orbital of Cu^2^^+^ (Figure S8) [40].

**FIGURE 3:**
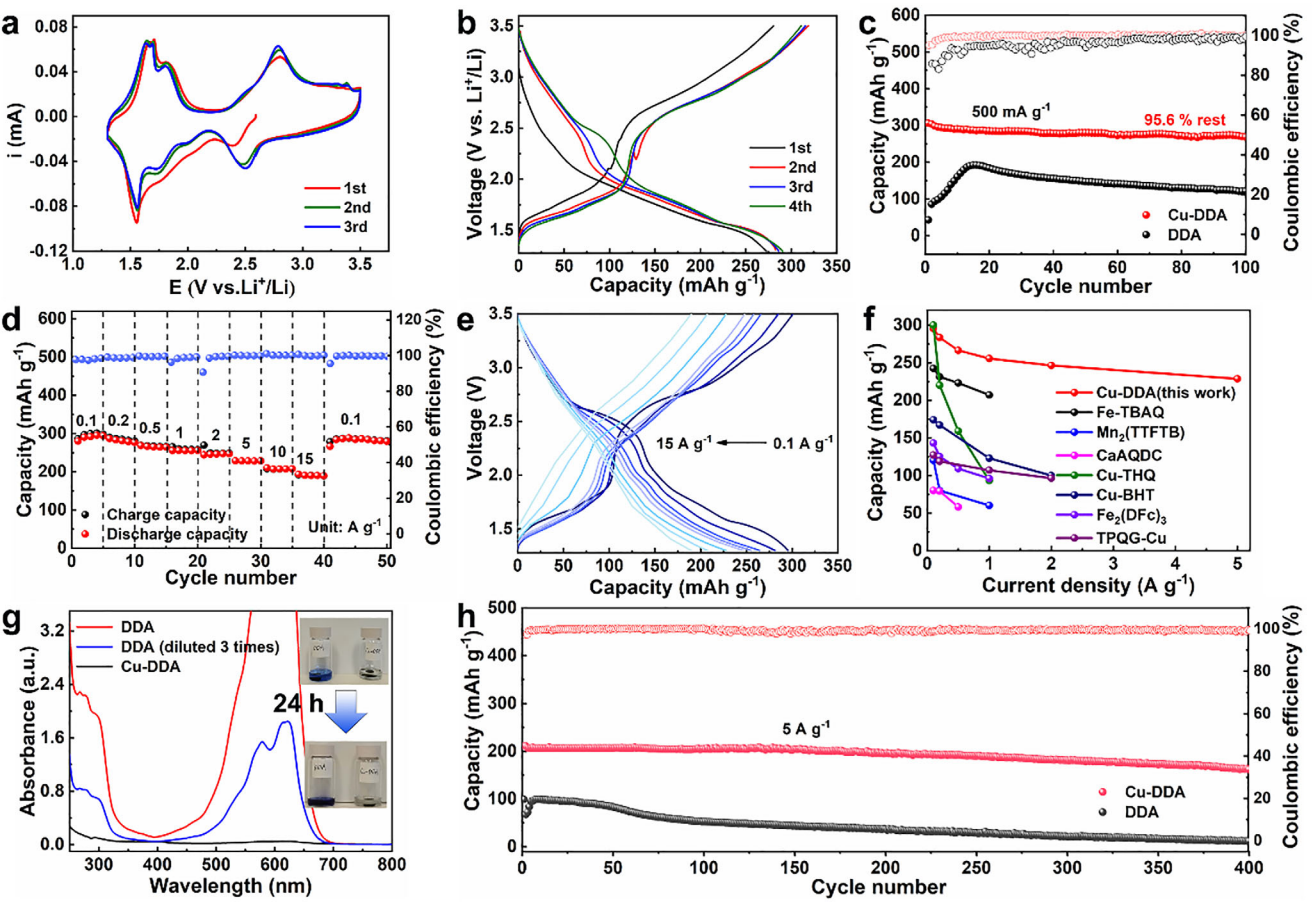
(a) CV curve at a scan rate of 0.1 mV s^−1^ between 1.3 and −3.5 V (vs. Li^+^/Li). (b) discharge/charge curve for the first four cycles and (c) related cycling stability test at 0.5 A g^−1^. (d) rate performance in the current density range of 0.1–15 A g^−1^. (e) discharge/charge curves at different current densities. (f) Comparison of rate capacities of the Cu‐DDA cathode and the reported MOP‐based cathodes. (g) The UV–vis‐NIR absorption for Cu‐DDA and DDA‐based cathodes in the electrolyte for 24 h. (h) Long‐term cycling performance at 5 A g^−1^.

The electrochemical performances of Cu‐DDA MOP were then assessed in coin cells with a potential range of 1.3–3.5 V (vs. Li^+^/Li). The corresponding cyclic voltammetry (CV) curves of the first three cycles are displayed in Figure 3a. The two pairs of oxidation/reduction peaks at 1.66/1.58 and 1.82/1.74 V are identified as the reversible reaction of C═O and C─O in the organic ligand [41]. Notably, the split oxidation peak at 1.66 V may originate from the reaction of the quinone group [42, 43]. The peaks at 2.48/1.99 V may belong to the redox of the imine group of DDA [35]. The couple of peaks at 2.8/2.51 V can be attributed to the reversible reaction between Cu^2+^ and Cu^+^ [44, 45].

The galvanostatic charge/discharge profiles (GCD) of Cu‐DDA MOP are tested at 500 mA g^−1^ (Figure 3b). Cu‐DDA MOP provides a high initial discharge/charge capacity of 272.7/280.3 mAh g^−1^. While the Coulombic efficiency fluctuates over the first ten cycles, this instability is attributed to irreversible electrolyte decomposition on Cu‐DDA framework and the gradual electrochemical activation of surface‐accessible Cu^2+^/Cu^+^ and C═O/C─O, C═N/C‐N redox sites [29, 46, 47]. Notably, the voltage platform between 2.0 and 2.8 V in the discharging process becomes increasingly significant and stabilized over time (Figure S9), which corresponds to the Cu^2+^ reduction process of the CV curve. The EIS test also confirms that the positive Cu‐DDA electrode is more active and shows less resistance after 10 cycles (Figure S10). The Cu‐DDA MOP retains 95.6% of its capacity after 100 cycles, with a Coulombic efficiency close to 100% (Figure 3c). The CV profile of the conductive carbon is tested in the same potential window, showing a SEI formation peak in the first cycle and no significant peaks in subsequent cycles. The pure conductive carbon exhibits a low capacity of approximately 15 mAh g^−1^ at a current density of 0.5 A g^−1^ (Figure S11), which has a small impact on the whole Cu‐DDA capacity.

Moreover, the Cu‐DDA exhibits outstanding rate performance, as displayed in Figure 3d,e. The Cu‐DDA cathode demonstrates stable capacities of 296, 284, 266, 256, 246, 229, 207, and 190 mAh g^−1^ at 0.1, 0.2, 0.5, 1.0, 2.0, 5.0, 10.0, and 15.0 A g^−1^, signifying its excellent rate performance. Upon returning the current density to 0.1 A g^−1^, the Cu‐DDA can deliver a capacity of 280 mAh g^−1^, which indicates a robust structural stability under rapid lithiation and delithiation conditions [48]. This observation aligns well with the Bader charge analysis, confirming efficient charge transfer processes during cycling. Importantly, these rate testing results surpass many reported MOP‐based cathode performance (Figure 3f) [35, 41, 47, 49, 50, 51, 52].

The solubility of Cu‐DDA was examined using UV–vis‐NIR absorption spectroscopy, revealing its remarkable insolubility in the electrolyte (i.e., LiTFSI/DOL/DME) [46, 53]. After 24 h, the Cu‐DDA electrode showed no detectable UV absorption, confirming the absence of dissolution. In contrast, the DDA electrode fully dissolved in the electrolyte, retaining significant UV absorption even after threefold dilution (Figure 3g). These results underscore the enhanced stability of Cu‐DDA in electrolyte solutions, suggesting that its MOP structure effectively mitigates solubility issues [29]. This stability can be attributed to two factors. One is the π‐d conjugation as revealed by the DOS analysis (Figure 1f), which reinforces structural integrity through electron delocalization within the organic ligands. The other is the high fracture toughness, which enables Cu‐DDA to prevent crack propagation during charge‐discharge cycles, thereby limiting the increase in surface area and consequently suppressing further dissolution.

Due to these merits, the Cu‐DDA‐based cathode maintains a high‐capacity retention, with 96.2% after 200 cycles and approximately 78% after 400 cycles at a high current density of 5 A g^−1^ (Figure 3h). Moreover, the Cu‐DDA electrode can still exhibit a high specific capacity of about 100.3 mAh g^−1^ at 5 A g^−1^ after 2100 cycles with a capacity degradation rate of only 0.027% per cycle (Figure S12). To further verify its practical fast‐charging viability, the Cu‐DDA cathode was also tested at a high rate with high mass loading (>3 mg cm^−2^). As shown in Figures S13 and S14, the Cu‐DDA cathode delivered a high capacity of 265 mAh g^−1^ and an areal capacity of about 0.56 mAh cm^−2^ at 500 mA g^−1^, corresponding to a cell capacity of about 0.6 mAh. Even at a high current density of 2 A g^−1^, it retained a capacity of 183 mAh g^−1^ with an areal capacity of ∼0.33 mAh cm^−2^, demonstrating robust fast‐charging capability under practically relevant electrode conditions. In contrast, DDA electrodes exhibit limited capacity and poor cycling stability, particularly under high current densities, where the capacity rapidly deteriorates. The coulombic efficiency of DDA exhibits severe fluctuation, dropping to as low as about 40% in the early cycles before gradually recovering, yet remaining unstable throughout the whole cycling process (Figure S15). This significant instability stems from the high solubility of DDA in the electrolyte and potential structural degradation during repeated redox processes. To further investigate the stability of Cu‐DDA under different extreme conditions, the electrochemical performance of Cu‐DDA cathode was evaluated at a low temperature of ‐30°C. The increased electrolyte viscosity at low temperature slowed surface wetting and prolonged the activation process, resulting in more pronounced initial Coulombic efficiency fluctuation. After activation, Cu‐DDA cathode delivered a stable discharge capacity of 190.4 mAh g^−1^ at 0.2 A g^−1^, and demonstrated excellent cycling stability over 250 cycles with almost 100% Coulombic efficiency (Figure S16). Regarding voltage window and cycling stability, the 1D Cu‐DDA demonstrates a high performance on par with that of 2D and 3D MOP‐based cathodes, even those with larger specific surface areas. (Table S6) [35, 41, 54].

**FIGURE 4:**
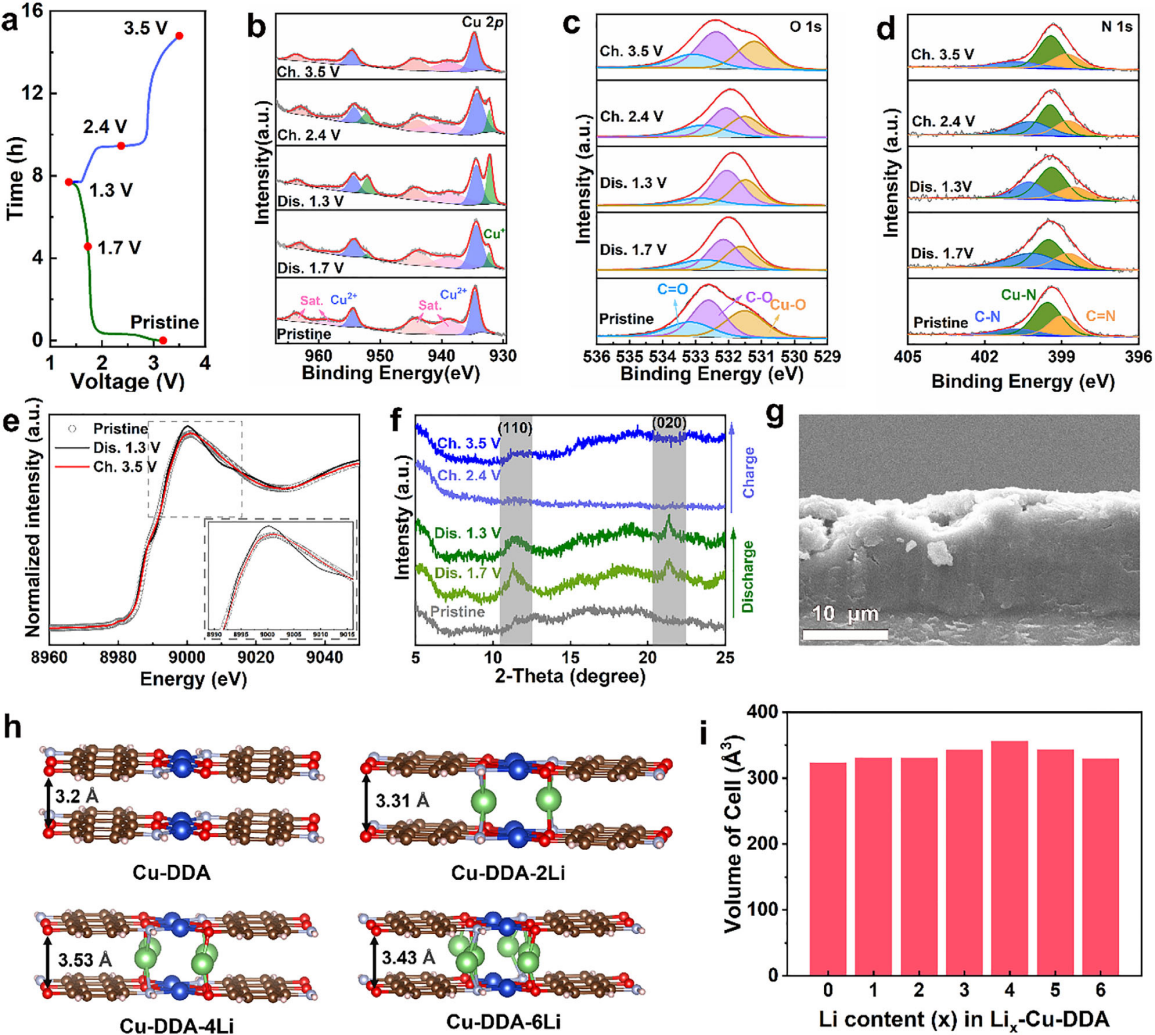
(a) Discharge‐charge profile of Cu‐DDA electrode at 50 mA g^−1^. (b–d)Ex situ XPS spectra of Cu‐DDA at Cu 2*p* (b), O 1s (c), and N 1s (d) at different discharge/charge states. (e) Ex situ EXAS spectrum of Cu‐DD A at pristine, charged, and discharged states. (f) Ex situ XRD of Cu‐DDA at different discharge/charge states. (g) SEM cross‐sectional images of the copper‐DDA electrode after cycling 400 cycles at 5 A g^−1^. (h) The change of layer spacing during the process of lithiation. (i) Cell volume changes of Cu‐DDA during Li^+^ insertion. (dis. means discharged, and ch. Means charged).

To investigate the lithiation/delithiation mechanism of the Cu‐DDA electrode during the battery process, diverse characterizations were carried out at different charging and discharging voltage points (Figure 4a). First, the XPS analysis was performed on the pristine and different charge/discharge states (Figure 4b–d). In the Cu 2*p* spectrum, the Cu^2^^+^ signals at 934.4 and 954.3 eV gradually attenuate during the discharging process, accompanied by the generation and progressive intensity of the Cu^+^ signals at 932.3 and 952.1 eV. During the subsequent charging phase, the behavior of the Cu^+^ peaks contrasts with that of the Cu^2+^ peaks, gradually recovering to their initial state, thereby demonstrating that the reversible redox behavior of Cu ions during the cycling process can provide favorable Li storage sites [29, 54]. Besides, as the process of delithiation goes on, the intensities of the C═O peak at 533.2 eV and C═N bond at 399 eV decrease, while the peaks corresponding to C─O at 532.6 eV and C─N bond at 400.7 eV increase. When lithiation, the relative densities of C═N, C─N, C═O, and C─O are restored to their initial levels, validating the high reversibility of the Cu‐DDA cathode during the lithiation and delithiation processes

To further reach out to the internal storage mechanistic insights of Cu‐DDA cathode, the FT‐IR spectrogram with different lithiation/delithiation states was illustrated (Figure S17). The peaks at 1510 cm^−1^, associated with the stretching vibration of the imine (C═N) group, and at 1615 cm^−1^, corresponding to the vibration frequency of the C═O group, weaken during discharge due to the formation of C─O bonds and the insertion of Li^+^ ions [35, 55]. It is suggested that results in the conversion of C═O and C═N groups to C─O─Li and C─N─Li bonds, which revert to C═O and C═N upon Li^+^ extraction during charging. These results are well matched with the electrostatic potential map of Cu‐DDA (Figure S18). The minima site of electrostatics is located on atom O and the atom N, indicating the Li ions tend to be absorbed by the atom N and the O atom [35].

To gain more insight into the electronic structure of copper, x‐ray absorption spectroscopy (XAS) measurements were performed (Figure 4e). When discharged to 1.3 V, the spectra shift slightly to the lower energy, indicating a reduction in the oxidation state of Cu. Conversely, recharging to 3.5 V restored the edge position to that of the pristine state, suggesting reversible redox behavior [41, 56].

Furthermore, ex situ XRD measurement was conducted to gain insights into the structure and crystalline (Figure 4f). During the charge and discharge process, the characteristic peaks of Cu‐DDA exhibited noticeable shifts. Specifically, the (110) peak at 11.3° moved to a smaller angle upon discharging to 1.7 V and further shifted to 11.3° at 1.3 V, indicating lattice expansion as Li^+^ ions intercalated into the structure. Upon charging to 3.5 V, the peak shifted back toward 11.3°, suggesting a partial recovery of the lattice structure. In addition, the peak representing the surface of (020) becomes increasingly obvious during the discharge process, which may be attributed to the enhanced structural ordering along the chain direction induced by Li^+^ insertion, as well as the partial interlayer hydrogen bonding due to the reduction of imine and carbonyl groups [35] The intensity of the (020) facet gradually decreases during charging, indicating the recovery of the crystal lattice. It is worth noting that these behaviors in the second cycle are similar to those of the first cycle (Figure S19), demonstrating the good crystal reversibility of Cu‐DDA in the lithiation/delithiation process.

SEM images show that after 400 cycles at 5 A g^−1^, Cu‐DDA exhibits almost no micro‐cracks and the surface is still smooth, supporting its high fracture toughness (Figure 4g, Figure S20). Moreover, the layer spacing expands during the Li^+^ insertion, suggesting the progressive exposure of active sites (Figure 4h) [55]. Remarkably, the volume of Cu‐DDA is maintained during the six Li^+^ cations insertion process (Figure 4i), attributed to the optimal balance between ductility and stiffness in Cu‐DDA. This balance allows for accommodating stress from volumetric fluctuations during Li^+^ insertion and prevents structural collapse or cracking while cycling. Overall, the unit cell volume of Cu‐DDA remains essentially unchanged with increasing Li‐ion concentration, highlighting its exceptional mechanical robustness and excellent cycling stability [31]. The redox activity of C─O/C═O, C─N/C═N groups and the Cu(I)/Cu(II) couple, combined with the high fracture toughness of the Cu‐DDA framework, enables the cathode to reversibly accommodate up to 6 Li^+^ ions during the electrochemical process (Figure S21).

**FIGURE 5:**
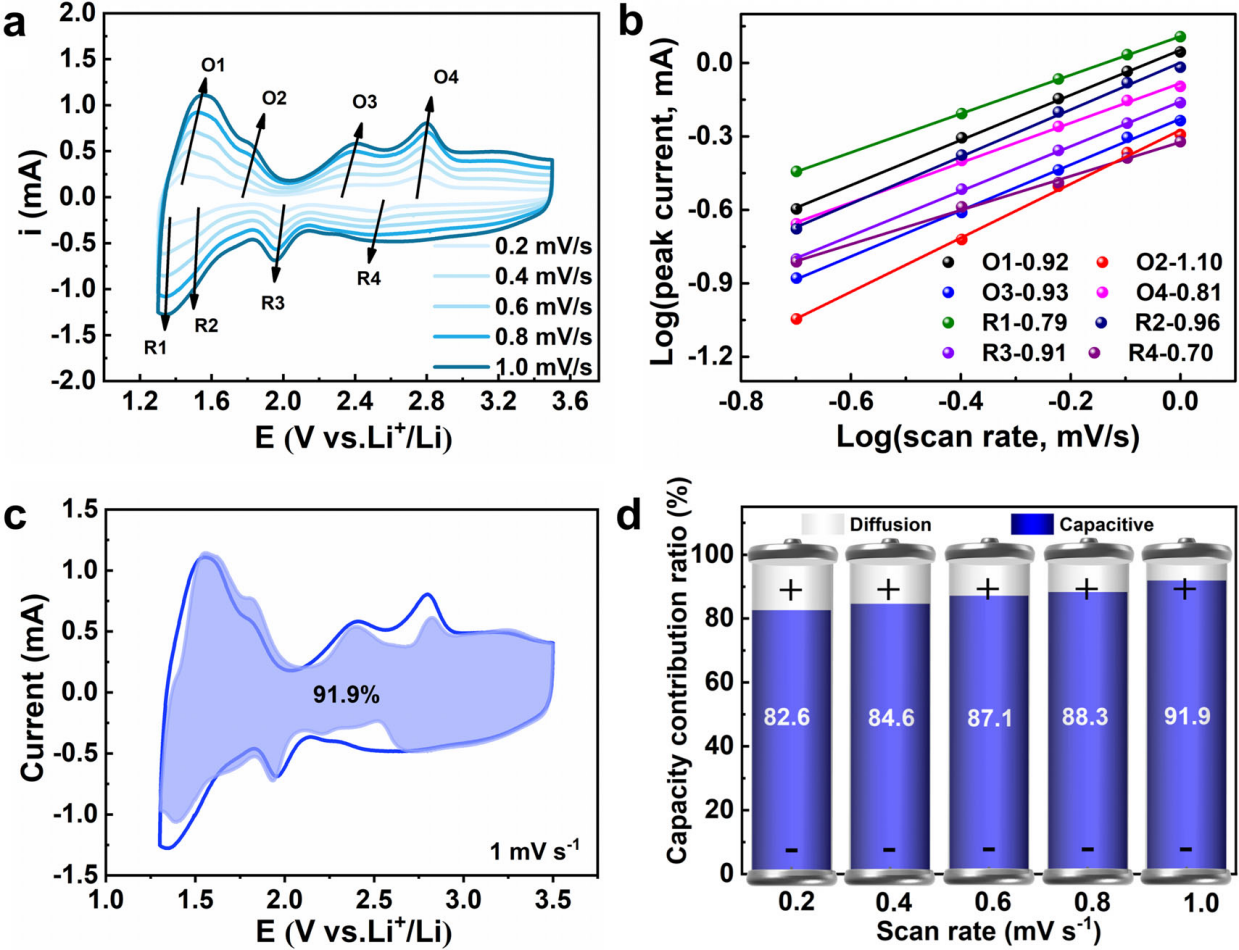
(a) CV curves of Cu‐DDA at different scan rates. (b) the b values determination of different peaks. (c) total current and current ration from capacitive‐controlled process at the scan rate of 1 mV s^−1^. (d) Percentage of capacitance contribution at various scan rates.

A better understanding of the kinetic behavior of Cu‐DDA is investigated via CV curves at different scan rates from 0.2 to 1.0 mV s^−1^. As the scan rate increases, the redox peaks shift, and the current increases (Figure 5a). The dynamic behavior of Li^+^ during the process can be evaluated by examining the relationship between the peak current (i) and the scan rate (v), using the equation [58]

(2) $$i={\mathrm{av}}^{b}$$

which is equivalent to log(i) = log(a) + blog(v). Among them, the b value of 0.5 indicates a Li^+^ ion diffusion‐controlled process, while a b value close to 1 signifies capacitive behavior [57, 58]. As shown in Figure 5b, the b value of O1/R1, O2/R2, and O3/R3 is close to 1, suggesting a capacitive‐controlled process in the voltage window between 1.3 and 2.6 V. Besides, the redox behavior of Cu^+^/Cu^2^^+^ involves both diffusion and capacitive contributions, as evidenced by the b‐values of 0.81/0.70 for O4/R4. Thus, the whole lithiation/delithiation process is governed by a combination of diffusion and capacitive mechanisms [35, 56]. Based on the following equation [59, 60]

(3) $$i\left(V\right)=k_{1}v+k_{2}v^{1/2}$$

where k_1_ and k_2_ are parameters obtained by fitting *iv* ^−^ *^1/2^* and v*^1/2^*. It is found that the capacitive contribution increases at increasing scan rate from 0.2 to 1.0 mV s^−1^, reaching 91.9% at 1.0 mV s^−1^ (Figure 5c,d). The high pseudocapacitance characteristics of the Cu‐DDA cathode can be attributed to its one‐dimensional π–d conjugated framework, in which the extended conjugation and exposed redox‐active Cu centers offer abundant accessible sites for Li^+^ adsorption and rapid charge transfer. In addition, the robust mechanical stability of the polymeric chains and the short diffusion pathways within the 1D channels further facilitate fast ion transport, collectively contributing to its remarkable rate capability. Such surface‐controlled redox reactions are less dependent on long‐range ion diffusion and therefore exhibit reduced temperature sensitivity compared with diffusion‐limited intercalation processes. Furthermore, the π‐d conjugated coordination framework provides efficient electron transport pathways, which help to maintain rapid charge transfer even under extreme conditions such as high current and low temperature. These combined factors contribute to the stable cycling performance and high coulombic efficiency observed at −30°C.

The diffusion coefficient of Li^+^ ions (D_Li_^+^) in Cu‐DDA cathode was calculated via the galvanostatic intermittent titration technique (GITT) measurement (Figure S22a). It is found that the Cu‐DDA cathode exhibits excellent kinetic properties with a remarkable Li^+^ diffusion coefficient of 10^−10^∼ 10^−11^ cm^2^ s^−1^ (Figure S22 b,c). Compared with traditional electrode materials, the Cu‐DDA cathode exhibits a competitive diffusivity performance (Table S7). The EIS analysis also supports that the Cu‐DDA cathode has a lower R_ct_ and Warburg factor (Figures S23 and S24), indicating the Li^+^ ion can diffuse more readily in the Cu‐DDA cathode [61]. These differences can be attributed to the rapid charge transfer by the electron delocalization of the π‐d conjugation [62, 63, 64].

## 3 Conclusion

In summary, a 1D π‐d conjugated metal–organic polymer is successfully synthesized by coordinating 1,5‐Diamino‐4,8‐dihydroxy‐9,10‐anthraceneedione (DDA) ligands with Cu^2+^ ion as the cathode for Lithium‐ion batteries. Coordinated with the π‐d conjugation, the resultant Cu‐DDA MOP displays a high reversible capacity of 272 mAh g^−1^ at 0.5 A g^−1^, a remarkable rate capacity with almost 100% Coulomb efficiency (207 and 190.2 mAh g^−1^ at 10 and 15 A g^−1^, respectively), and outstanding long‐term cycling (up to 400 cycles at 5 A g^−1^). By the verification of FT‐IR, XPS, and XRD combined with DFT computations, we can attribute the excellent performances to the dual redox activities of the Cu^2+^ center and DDA ligands. More importantly, the high fracture toughness of Cu‐DDA plays a pivotal role in enhancing its mechanical robustness for fast charging. It can also effectively mitigate structural degradation under high current densities, ensuring long‐term cycling stability. These results suggest that the high fracture toughness of Cu‐DDA plays an important role in its fast‐charging stability, highlighting mechanical performance as a potentially important design consideration for metal‐organic polymer‐based fast‐charging electrodes.

## Funding

This work was funded by Australian Research Council Discovery Project (Grant No. DP210103266, DP210104010). National Computational Infrastructure (Grant No. P74).

## Conflicts of Interest

The authors declare no conflicts of interest.

## Supporting information




**Supporting File**: advs75786‐sup‐0001‐SuppMat.docx.

## Data Availability

The data that support the findings of this study are available within the article and its supplementary material
