# Supplementary material for: High Fracture Toughness of 1D Copper‐Based MOP Electrode Enables Fast‐Charging Lithium‐Ion Batteries
Source: Adv Sci (Weinh). 2026 May 22;13(46):e75786. doi: 10.1002/advs.75786 (PMC13335880; doi:10.1002/advs.75786)
Supplement: Supplementary file 1 [file ADVS-13-e75786-s001.docx]

Supporting Information

**High Fracture Toughness of 1D Copper-based MOP electrode Enables Fast-Charging Lithium-ion Batteries**

Mingli Li, Zhenzhen Wu^*^, Pan Yang, Di Zhao, Mengyang Dong, Muhammad Tayyab Ahsan, Lei Zhang, Shanqing Zhang^*^, Yun Wang^*^

Mingli Li, Zhenzhen Wu, Pan Yang, Di Zhao, Mengyang Dong, Yun Wang

School of Environment and Science, Griffith University, Gold Coast Campus, Southport 4222, Australia

E-mail: yun.wang@griffith.edu.au, zhenzhen.wu@griffith.edu.au

Muhammad Tayyab Ahsan, Shanqing Zhang,

Institute for Sustainable Transformation, School of Chemical Engineering and Light Industry, Guangdong University of Technology, Guangzhou 510006, China.

E-mail: s.zhang@gdut.edu.cn

**1. Experimental Section**

**1.1 Chemicals and materials**

1,5-Diamino-4,8-dihydroxy-9,10-anthraceneedione (DDA, 95%) was obtained from Aladdin Co. Ltd., Copper acetate ((CH_3_COO)_2_Cu, 90%) was purchased from Aladdin Co. Ltd., ethanol (CH_3_CH_2_OH,) was obtained from Chem. Supply Pty Ltd. multi-walled carbon nanotubes (CNT), was purchased from Shanghai Hushi Co., Ltd. Bis(trifluoromethane)sulfonimide lithium salt (LiTFSI), 1,3-Dioxolane (DOL), and 1,2-Dimethoxyethane (DME), N-methyl pyrrolidone (NMP) were purchased from Sigma Aldrich.

**1.2 Synthesis of Cu-DDA**

According to previous method,^[1]^ 55.5 mg (CH_3_COO)_2_Cu and 75 mg DDA were dissolved in 400 mL ethanol and 100 mL deionized water, respectively. After that, dropping the (CH_3_COO)_2_Cu solution into the DDA solution with the speed of 2 drop per second. Then, the mixed solution was transferred to 1000 mL flask and was reacted at 90 ℃ for 24 h. After cooling at room temperature, the precipitate was washed several times with ethanol and deionized water. Finally, the sediment was dried in a vacuum drying oven at 60 °C for 12 h.

**1.3 Characterization Methods**

The crystallographic structure was characterized by powder X-ray diffraction instrument (pXRD) with Cu Kα radiation (λ=1.5406 Å). N_2_ adsorption-desorption isotherms and pore size distribution were measured on a Micromeritics ASAP 2020 at 77 K and a pressure of 1 bar. The morphologies and structure were inspected by scanning electron microscopy (SEM) and the transmission electron microscopy (TEM). Fourier transform infrared (FT-IR) spectra was obtained by the Bruker Alpha I spectrometer. X-ray photoelectron spectroscopy (XPS) was performed using a VG ESCALAB250 device and corrected with C 1s level at 284.8 eV. Electron paramagnetic resonance (EPR) was investigated by a Bruker E580-10/12 spectrometer. Ultraviolet-visible-near-infrared (UV-vis-NIR) spectrum was collected on the Agilent Cary 5000. Cu K-edge X-ray absorption spectra were collected on the hard X-ray beamline at the Australian Synchrotron (Melbourne, Australia). The extended X-ray absorption fine structure (EXAFS) analyses were conducted via the ATHENA module. Fourier transform of the *k*^2^-weighted EXAFS oscillations was used to evaluate the contribution of each bond to the Fourier transform peak.

**1.4 Electrochemical Measurement**

Electroactive materials (Cu-DDA or DDA) were mixed with carbon nanotubes and polyvinylidene fluoride (PVDF) binder in a mass ratio of 50:40:10, using N-methyl-2-pyrrolidone (NMP) as the solvent to prepare the working electrodes. The slurry was ground in an agate mortar for 30 minutes and then uniformly applied to the aluminum foil. After drying for 12 hours in a vacuum oven at 60℃, the electroactive coated aluminum foil is drilled into a small disk with a diameter of 1.1 cm, and the surface load of the active substance is concentrated in the range of 0.7 to 1.0 mg cm^−2^. Electrochemical testing was measured on CR2032 coin cells with a Lithium metal anode, the polypropylene was used as the separator, and 1.0 M LiTFSI/DOL/DME (vol% 1:1) as the electrolyte in a glovebox at Ar-atmosphere (both O_2_ and H_2_O <0.1 ppm). Galvanostatic charge/discharge profiles and corresponding cycling testing were investigated by Neware-CT-4008T battery instrument (Neware Co., Ltd., Shenzhen China) in the voltage range of 1.3 - 3.5 V. CV and EIS measurements were tested on the electrochemical workstation Biologic SP-200. The GITT testing was conducted with a pulse current of 50 mA g^-1^ for 15 minutes and a relaxation period of 60 minutes between pulses, followed by the first cycled one time under galvanostatic charge-discharge at 0.05 A g^-1^.

The Li^+^ diffusion coefficient D_Li_^+^ can be calculated via the following equation^[2-3]^.

$\mathbf{D}_{\mathbf{Li}}\mathbf{+ =}\frac{\mathbf{4}}{\boldsymbol{\pi}\boldsymbol{\tau}}{\mathbf{(}\frac{\mathbf{m}_{\mathbf{B}}\mathbf{V}_{\mathbf{M}}}{\mathbf{M}_{\mathbf{B}}\mathbf{S}}\mathbf{)}}^{\mathbf{2}}{\mathbf{(}\frac{\boldsymbol{\Delta}\mathbf{E}_{\mathbf{s}}}{\boldsymbol{\Delta}\mathbf{E}_{\boldsymbol{\tau}}}\mathbf{)}}^{\mathbf{2}}$	(S1)

where τ is the duration of the current pulse, and m_B_, V_M_, and M_B_ represent the mass loading, molar volume, and molar mass of the electrode active material, respectively. S refers to the electrode–electrolyte contact area.ΔE_S_ corresponds to the voltage difference at steady state between each adjacent step, and ΔE_τ_ is the voltage change induced by each current pulse.

**1.5 Theoretical methods**

All density functional theory (DFT) simulations were performed via the Vienna ab initio simulation package (VASP) with the use of the projector-augmented wave (PAW) method.^[4]^ The electron-correlation was accounted for using the generalized gradient approximation (GGA) with the Perdew–Burke–Ernzerhof (PBE) functional^.[5]^ The plane-wave energy cutoff was set to 520 eV, the convergence criteria of the total energy and force were set to 10^-5^ eV and 0.02 eV Å^-1^. For Correcting the van der Waals interaction, the DFT-D3 method was applied. Owing to the strong on-site Coulomb repulsion between the d electrons of Cu atoms, the DFT + U method with U = 4.0 eV was used to describe the electronic properties of Cu-DDA.^[6]^ The structures of Cu-DDA and lithiated Cu-DDA were optimized by minimizing the energies of the 1 × 1 × 1 bulk unit cell using a k-point mesh of 2 × 3 × 8.^[7]^ All the results were further processed by vaspkit.^[8]^

The Bulk modulus and Shear modulus were calculated by VASP in MedeA with the convergence criteria of the total energy of 10^-7^ eV with the strain of 0.01-10%. The bulk modulus (B_V_) and shear modulus (G_V_) via Viogt method are calculated by the following equations: ^[9]^

$B_{V}=\frac{1}{9}\left[ {(C}_{11}+C_{22}+C_{33})+2{(C}_{12}+C_{23}+C_{31}) \right]$	(S2)

$G_{V}=\frac{1}{15}\left[ {(C}_{11}+C_{22}+C_{33})-{(C}_{12}+C_{23}+C_{31})+3{(C}_{44}+C_{55}+C_{66}) \right]$	(S3)

where C_ij_ is the elastic constants.

And the bulk modulus (B_R_) and shear modulus (G_R_)via Reuss method is obtained by the following equations: ^[10]^

$\frac{1}{B_{R}}={(S}_{11}+S_{22}+S_{33})+2{(S}_{12}+S_{23}+S_{31})$	(S4)

$\frac{1}{G_{R}}=\frac{1}{15}\left[ {4(S}_{11}+S_{22}+S_{33})-{4(S}_{12}+S_{23}+S_{31})+3{(C}_{44}+C_{55}+C_{66}) \right]$	(S5)

where Sij are the compliance constants, calculated from the inverse matrix of the stiffness matrix C_ij._

To get a better approximation of elastic modulus, the final bulk modulus (B) and shear modulus (G) can be obtained by the Voigt-Reuss-Hill approximation with the following equations: ^[11]^

$B=\frac{1}{2}(B_{V}+B_{R})$	(S6)

$G=\frac{1}{2}(G_{V}+G_{R})$	(S7)

Due to the absence of direct experimental mechanical validation, the correlation between K_IC_ values predicted by DFT and electrochemical performance can only be regarded as a correlation result. To validate our method, we used LiCoO_2_ as a model system. LiCoO_2_ is a classical cathode material with well-established mechanical data. Our calculated K_IC_ value of LiCoO_2_ is 1.35 MPa·m^1/2^ via using the same DFT parameters and Equation (1). This result is in excellent agreement with the experimentally reported values in the literature(1.7 ± 0.4 MPa·m^1/2^). ^[12]^

To justify the applicability of Equation (1) for facture thoughness for evaluating our 1D MOPs, we employed graphite as a structural benchmark since graphite shares fundamentally similar chemo-mechanical features with our 1D π- conjugated MOPs. Both of them are highly anisotropic low-dimensional systems characterized by strong intra-layer/intra-chain bonds and weak vdW inter-layer/inter-chain interactions. In such materials, the dominant intrinsic fracture mode under stress is typically the cleavage along the weakest non-covalent planes.

The exact same computational methodology and Equation (1) were used to calculate the K_IC_ of graphite. The bulk modulus and shear modulus of graphite are 23.2 GPa and 235.9 GPa, respectively.^[13]^ The calculated K_IC_ is 1.06 MPa m^1/2^. The experimental K_IC_ of graphite is tested in a range of 0.82 to 1.27 MPa m^1/2^. ^[14]^ The calculated result is in great agreement with the experimental result. It demonstrates that Equation (1) from the covalent/ionic solids can be applied to low-dimensional materials, thereby demonstrating its validity for evaluating our 1D MOPs.

**Figure S1.** Four-point probe conductivity testing for Cu-DDA.

**Figure S2.** Bandgap comparison for Cu-DDA and DDA.

`

**Figure S3.** HOMO and LUMO plot for Cu-DDA unit

**Figure S4.** HOMO and LUMO plot for DDA unit.

**Figure S5.** (a) N_2_ absorption/desorption isotherms. (b) pore size distribution profile of Cu-DDA.

**Figure S6.** Cu K-edge spectrum of EXAFS curves of Cu-DDA, Cu, Cu_2_O, and CuO.

**Figure S7.** (a) XPS full summary of Cu-DDA and DDA. High-resolution XPS spectra of Cu-DDA and DDA in (b) Cu 2p, (c) O 1s and (d) N 1s.

**Figure S8.** EPR spectrum of Cu-DDA powder.

**Figure S9.** galvanostatic charge/discharge profiles of Cu-DDA at 0.5 A g^-1^.

**Figure S10.** EIS result for pristine and after 10 cycles testing at 0.5 A g^-1^.

**Figure S11.** (a) CV plot of pure CNT electrode (CNT: PVDF=9:1). (b) The cycling performance of pure CNT electrode.

**Figure S12.** Long cycling testing of Cu-DDA cathode at 5A g^-1^.

**Figure S13.** Cycling performance at a current density of 500 mA g^-1^ of Cu-DDA cathode with the high mass loading (3.51 mg cm^-2^ with the diameter of 11 mm and the thickness of 500 μm) presented using (a) specific capacity, (b) areal capacity.

**Figure S14.** Cycling performance at a high current density of 2 A g^-1^ of Cu-DDA cathode with high mass loading (3.70 mg cm^-2^ with the diameter of 11 mm and the thickness of 500 μm) presented using presented using (a) specific capacity, (b) areal capacity.

**Figure S15.** Coulombic efficiency of Cu-DDA and DDA at 5 A g^-1^, respectively.

**Figure S16**. Long cycling testing of Cu-DDA cathode and related coulombic efficiency of Cu-DDA at -30 °C.

**Figure S17.** Ex situ FT-IR testing in the first cycling test of Cu-DDA electrode at 50 mA g^-1^.

**Figure S18.** The electrostatic potential map of Cu-DDA at the 0.05 e bohr^-3^ charge density isosurface.

**Figure S19.** Ex-situ XRD testing of Cu-DDA electrode at 50 mA g^-1^.

**Figure S20.** SEM cross-sectional images of the copper-DDA electrode before cycling.

**Figure S21.** Proposed Li^+^ storage mechanism in Cu-DDA via a six-electron transferring process.

**Figure S22.** a) GITT curves and b, c) the calculated lithium-ion diffusion coefficients of Cu-DDA in the discharge and charge process, respectively.

**Figure S23.** EIS comparison between Cu-DDA and DDA electrode.

**Figure S24.** The connection between the impedance (Z') and the reciprocal of the square root of the frequency (ω^-0.5^).

The fitting process is conducted according to the expression formula of AC impedance:

𝑍 = 𝑅_𝑐𝑡_ + 𝑅_𝑠_ + 𝜎𝜔^-0.5^	(7)

Where Rct refers to charge transfer resistance, Rs refers to the diffusion impedance,σ refers to the Warburg factor, and ω refers to the angular frequency. Thus σ is correlate with the slope.

**Table S1.** Elastic constant matrix C_ij_ of DDA (unit: GPa)

| j  i | 1 | 2 | 3 | 4 | 5 | 6 |
| --- | --- | --- | --- | --- | --- | --- |
| 1 | 463.98 | -50.26 | -1.81 | 0.00 | 0.01 | -1.86 |
| 2 | -50.26 | 167.39 | -1.98 | 0.00 | 0.01 | -35.35 |
| 3 | -1.81 | -1.98 | 4.81 | -0.01 | 0.02 | 0.12 |
| 4 | 0.00 | 0.00 | -0.01 | -1.37 | 0.08 | 0.00 |
| 5 | 0.01 | 0.01 | 0.02 | 0.08 | -1.54 | 0.00 |
| 6 | -1.86 | -35.35 | 0.12 | 0.00 | 0.00 | 180.70 |

**Table S2.** Elastic compliance matrix S_ij_ of DDA(unit:10^-3^ 1/GPa)

| j  i | 1 | 2 | 3 | 4 | 5 | 6 |
| --- | --- | --- | --- | --- | --- | --- |
| 1 | 2.24 | 0.72 | 1.14 | 0.00 | 0.02 | 0.16 |
| 2 | 0.72 | 6.49 | 2.92 | -0.02 | 0.06 | 1.28 |
| 3 | 1.14 | 2.92 | 209.73 | -0.64 | 2.37 | 0.45 |
| 4 | 0.00 | -0.02 | -0.64 | -731.88 | -35.65 | 0.00 |
| 5 | 0.02 | 0.06 | 2.37 | -35.65 | -651.06 | 0.01 |
| 6 | 0.16 | 1.28 | 0.45 | 0.00 | 0.01 | 5.79 |

**Table S3.** Elastic constant matrix C_ij_ of Cu-DDA (unit: GPa)

| j  i | 1 | 2 | 3 | 4 | 5 | 6 |
| --- | --- | --- | --- | --- | --- | --- |
| 1 | 373.29 | 38.96 | -46.82 | 32.47 | -3.58 | -9.59 |
| 2 | 38.96 | 548.59 | -57.67 | 31.05 | -3.54 | -10.51 |
| 3 | -46.82 | -57.67 | -39.30 | 31.28 | -3.69 | -11.98 |
| 4 | 32.47 | 31.05 | 31.28 | -0.88 | 0.00 | 0.14 |
| 5 | -3.58 | -3.54 | -3.69 | 0.00 | -1.26 | -0.01 |
| 6 | -9.59 | -10.51 | -11.98 | 0.14 | -0.01 | 187.26 |

**Table S4.** Elastic compliance matrix S_ij_ of Cu-DDA(unit:10^-3^ 1/GPa)

| j  i | 1 | 2 | 3 | 4 | 5 | 6 |
| --- | --- | --- | --- | --- | --- | --- |
| 1 | 2.44 | -0.41 | -2.11 | 0.27 | 0.43 | -0.03 |
| 2 | -0.41 | 1.67 | -1.21 | 0.95 | 0.00 | -0.01 |
| 3 | -2.11 | -1.21 | 4.29 | 31.62 | -3.11 | 0.07 |
| 4 | 0.27 | 0.95 | 31.62 | 31.33 | -96.13 | 2.06 |
| 5 | 0.43 | 0.00 | -3.11 | -96.13 | -786.21 | -0.15 |
| 6 | -0.03 | -0.01 | 0.07 | 2.06 | -0.14 | 5.34 |

**Table S5.** Mechanical parameter comparison of Cu-DDA and traditional electrode materials.

| **Materials** | **K_IC_ (MPa m^1/2^)** | **Ref.** |
| --- | --- | --- |
| **Cu-DDA** | **2.50** | **This work** |
| LiCoO_2_ | 1.1 | ^[12]^ |
| LiMn_2_O_4_ | 0.24 | ^[15]^ |
| LiNi_0.5_Mn_0.3_Co_0.2_O_2_ | 0.30 | ^[16]^ |
| LiNi_0.5_Co_0_Mn_0.5_O_2_ | 1.50 | ^[17]^ |
| LiNi_0.6_Mn_0.2_Co_0.2_O_2_ | 0.26 | ^[18]^ |
| LiNi_0.8_Co_0.15_Al_0.05_O_2_ | 1.48 | ^[17]^ |

**Table S6.** Electrochemical performance of MOP-based cathode in Lithium-ion batteries

| **Materials** | **Dimension** | **Working voltage** | **Capacity** | **Cycling stability** | **Battery type** | **Ref.** |
| --- | --- | --- | --- | --- | --- | --- |
| **Cu-DDA** | **1D** | **1.3-3.5 V** | 190 mAh g^-1^  at 15000 mA g^-1^ | **78%**  **at 5 A g^-1^**  **after 400 cycles** | **LIB** | **This work** |
| [CuL(DMF)_2_]_n_ | 1D | 1.5-3.6V | 268 mAh g^-1^  at 30 mA g^-1^ | 120 mAh g^–1^  at 80 mA g^–1^  after 33 cycles | LIB | ^[19]^ |
| Cu-DHAQ | 1D | 1.5−3.6 V | 115 mAh g^-1^  at 50 mA g^-1^ | 96.5%  at 50 mA g^–1^  after 100 cycles | LIB | ^[20]^ |
| Co-DTBPT | 1D | 1.5−4.0 V | 71.6 mAh g^−1^  at 50 mA g^−1^ | 55 mA h g^−1^  at 50 mA g^−1^  after 50 cycles | LIB | ^[21]^ |
| Fe-TABQ | 2D | 1.3-3.6 V | 206 mAh g^–1^  at 1000 mA g^–1^ | 214.6 mAh g^–1^  at 800 mA g^–1^  after 200 cycles | LIB | ^[22]^ |
| Cu-THQ | 2D | 1.2-4 V | 387 mAh g^–1^  at 50 mA g^–1^ | 340 mAhg^-1^  at 50 mA g^–1^  after 100 cycles | LIB | ^[23]^ |
| Ni-TABQ | 2D | 1.0-3.5 V | 318.7 mAh g^-1^  at 50 mA g^-1^ | 236.7 mAhg^-1^  at 200 mA g^–1^  after 200 cycles | LIB | ^[24]^ |
| Cu-TPQG | 2D | 1.3-3.8 V | 150.2 mAh g^-1^  at 20 mA g^-1^ | 88.38 mAhg^-1^  at 1000 mA g^–1^  after 500 cycles | LIB | ^[25]^ |
| NiDI | 2D | 2.0-4.5 V | 155 mAh g^−1^  at 10 mA g^−1^ | 50%  at 300 mA g^–1^  after 250 cycles | LIB | ^[26]^ |
| Co_0.56_Ni_0.44_-HAB | 2D | 1.0–3.5 V | 248 mAh g^−1^  at 100 mA g^−1^ | ∼200 mAhg^-1^  at 100 mA g^–1^  after 20 cycles | LIB | ^[27]^ |
| Cu(2,7-AQDC) | 2D | 1.7-4.0 V | 147 mAh g^−1^  at 10 mA g^−1^ | 105 mAh g^-1^  at 200 mA g^–1^  after 50 cycles | LIB | ^[28]^ |
| HHB-Cu | 2D | 1.3-2.6 V | 153 mAh g^-1^  at 100 mA g^–1^ | 90%  at 1000 mA g^–1^  after 1000 cycles | LIB | ^[29]^ |
| Cu_3_(HHTP)_2_ | 2D | 1.7–3.5 V | ~ 105 mAh g^–1^  at 1 C | 95 mA·h·g^−1^  after 60 cycles | LIB | ^[30]^ |
| Cu_3_(HOTAT)_2_ | 2D | 1.7-3.5 V | 225 mAh g^–1^  at 0..01 A g^-1^ | 73% over 270 cycles at 0.2 A g^-1^ | LIB | ^[31]^ |
| Cu–BHT | 2D | 1.5–3.0 V | 232 mA·h·g^−1^  at 300 mA g^-1^ | 0.048% per cycle  at 300 mA g^–1^  upon 500 cycles | LIB | ^[32]^ |
| Fe_2_(DFc)_3_ | 3D | 2-4.2 V | 172 mAh g^-1^  at 50 mA g^-1^ | 70 mAh g^-1^  at 2000 mA g^-1^  for 10,000 cycles | LIB | ^[33]^ |
| (NBu_4_)_2_Fe_2_(DHBQ)_3_ | 3D | 1.5-3.5 V | 137.2 mAh g^-1^ at 10 mA g^-1^ | 103.1 mAh g^-1^  at 500 mA g^-1^  for 350 cycles | LIB | ^[34]^ |
| Fe_2_(DHBQ)_3_ | 3D | 1.5-3.8 V | 346 mAh g^−1^  at 50 mA g^−1^ | 285 mAh g^-1^  at 50 mA g^-1^  for 50 cycles | LIB | ^[35]^ |
| Co_2_(TTFTB) | 3D | 1.5-4.2 V | 81 mAh g^−1^  at 100 mA g^−1^ | 43 mAh g^-1^  at 100 mA g^-1^  after 30 cycles | LIB | ^[36]^ |
| Cu-TCA | 3D | 1.4- 4.3V | 102.2 mAh g^−1^  at 0.5C | 96.5% at a 2 C rate after 200 cycles | LIB | ^[37]^ |
| MIL-53(Fe) | 3D | 1.5-3.5V | 70 mAh g^−1^  at C/40 | 80%  at C/40 after 1hour | LIB | ^[38]^ |

**Table S7.** Comparison of Li ion diffusivity of Cu-DDA and traditional electrode materials.

| **Material** | **Li ion diffusivity *(*cm^2^ s^-1^）** | **Ref.** |
| --- | --- | --- |
| **Cu-DDA** | **10^-10^ to 10^-11^** | **This work** |
| LiFePO_4_ | 1.65 × 10^-11^ | ^[39]^ |
| NMC811 | 10^-7^ to 10^-11^ | ^[40]^ |
| lanthanum and aluminum LiCoO_2_ | 4.7 × 10^-11^ to 1.2 × 10^-10^ | ^[41]^ |
| Graphite | 6.51 × 10^-11^ to 1.12 × 10^-10^ | ^[42]^ |

**Reference**

[1]	S. Shang, C. Du, Y. Liu, et al., “A one-dimensional conductive metal-organic framework with extended pi-d conjugated nanoribbon layers,” *Nat. Commun.* 13, no. 1 (2022): 7599,

https://doi.org/10.1038/s41467-022-35315-0.

[2]	M. Qi, L. Cheng, X. Zhang, et al., “Two dimensional Conjugated Metal-Organic Frameworks with Multiple Redox-Active Sites towards High-Performance Sodium-Ion Battery,” *Adv. Sci.* (2025): 2503369,

https://doi.org/10.1002/advs.202503369.

[3]	X. Su, L. Cheng, X. Yan, et al., “In Situ Construction of Amide-Functionalized 2D Conjugated Metal-Organic Frameworks with Multiple Active Sites for High-Performance Potassium-Ion Batteries,” *J. Am. Chem. Soc.* 147,no. 21 (2025): 18338,

https://pubs.acs.org/doi/10.1021/jacs.5c07158.

[4]	W. Kohn, L. J. Sham, “Self-Consistent Equations Including Exchange and Correlation Effects,” *Phys. Rev.* 140, no. 4 (1965): A1133,

https://doi.org/10.1103/PhysRev.140.A1133.

[5]	K. B. J. P. Perdew, M. Ernzerhof,, “Generalized Gradient Approximation Made Simple,” *Phys. Rev. Lett.* 77, no. 18 (1996): 3865*.*

https://doi.org/10.1103/PhysRevLett.77.3865

[6]	S. Grimme, “Semiempirical GGA‐type density functional constructed with a long‐range dispersion correction,” *J. Comput. Chem.* 27, no. 15 (2006): 1787,

https://doi.org/10.1002/jcc.20495.

[7]	R. A. Evarestov, V. P. Smirnov, “Modification of the Monkhorst-Pack special points meshes in the Brillouin zone for density functional theory and Hartree-Fock calculations,” *Phy. Rev. B* 70, no. 23 (2004): 233101,

https://doi.org/10.1103/PhysRevB.70.233101.

[8]	V. Wang, N. Xu, J.-C. Liu, et al., “VASPKIT: A user-friendly interface facilitating high-throughput computing and analysis using VASP code,” *Comput. Phys. Commun.* 267 (2021): 108033,

https://doi.org/10.1016/j.cpc.2021.108033.

[9]	W. Voigt, Lehrbuch der kristallphysik:(mit ausschluss der kristalloptik), Vol. 34, BG Teubner, 1910.

[10]	A. Reuss, “Berechnung der fließgrenze von mischkristallen auf grund der plastizitätsbedingung für einkristalle,” Z. *Angew. Math. Mech.* 9, no. 49 (1929),

https://doi.org/10.1002/zamm.19290090104

[11]	R. Hill, “The elastic behaviour of a crystalline aggregate,” *Proc. Phys. Soc. A* 65, no. 5 (1952): 349,

https://doi.org/10.1088/0370-1298/65/5/307.

[12]	M. Qu, W. H. Woodford, J. M. Maloney, et al., “Nanomechanical Quantification of Elastic, Plastic, and Fracture Properties of LiCoO_2_,” *Adv. Energy Mater.* 2, no. 8 (2012): 940,

https://doi.org/10.1002/aenm.201200107.

[13]	I. Mosyagin, D. Gambino, D. G. Sangiovanni, et al., “Effect of dispersion corrections on ab initio predictions of graphite and diamond properties under pressure,” *Phys. Rev. B* 98, no. 17 (2018),

https://doi.org/10.1103/PhysRevB.98.174103

[14]	H. Wang, L. Sun, H. Wang, et al., "An experimental study on fracture toughness of a fine-grained isotropic graphite." in *Proceedings of the American Society of Mechanical Engineers*, vol. 44960 (2012): 175.

[15]	M. Z. Mughal, H. Y. Amanieu, R. Moscatelli, M. Sebastiani, “A Comparison of Microscale Techniques for Determining Fracture Toughness of LiMn_2_O_4_ Particles,” *Materials* 10, no. 4 (2017): 403,

https://doi.org/10.3390/ma10040403.

[16]	L. S. de Vasconcelos, N. Sharma, R. Xu, K. Zhao, “In-Situ Nanoindentation Measurement of Local Mechanical Behavior of a Li-Ion Battery Cathode in Liquid Electrolyte,” *Exp. Mech.* 59, no. 3 (2019): 337-347,

https://doi.org/10.1007/s11340-018-00451-6.

[17]	L. Xiao, “Mechanical properties modeling of cathode materials for lithium-ion batteries based on bond valence model,” *Mater.* *Today Commun.* 39, (2024): 108852,

https://doi.org/10.1016/j.mtcomm.2024.108852.

[18]	N. Sharma, D. Meng, X. Wu, L. S. de Vasconcelos, L. Li, K. Zhao, “Nanoindentation measurements of anisotropic mechanical properties of single crystalline NMC cathodes for Li-ion batteries,” *Extreme Mech. Lett.* 58 (2023): 101920,

https://doi.org/10.1016/j.eml.2022.101920.

[19]	C.-H. Chang, A.-C. Li, I. Popovs, et al., “Elucidating metal and ligand redox activities of a copper-benzoquinoid coordination polymer as the cathode for lithium-ion batteries,” *J. Mater. Chem. A* 7, no. 41 (2019): 23770-23774,

https://doi.org/10.1039/c9ta05244e.

[20]	Y. Wu, M. Lai, J. Liang, et al., “Advanced 1D Metal-Organic Coordination Polymer for Lithium-Ion Batteries: Designing, Synthesis, and Working Mechanism,” *ACS Appl. Mater. Inter.* 15, no. 1 (2022): 1452-1462,

https://doi.org/10.1021/acsami.2c20385.

[21]	D. Shen, Y. Sha, C. Chen, et al., “A one-dimensional cobalt-based coordination polymer as a cathode material of lithium-ion batteries,” *Dalton Trans.* 52, no. 21 (2023): 7079-7087,

https://doi.org/10.1039/d3dt00398a.

[22]	J. Geng, Y. Ni, Z. Zhu, et al., “Reversible Metal and Ligand Redox Chemistry in Two-Dimensional Iron-Organic Framework for Sustainable Lithium-Ion Batteries,” *J. Am. Chem. Soc.* 145, no. 3 (2023): 1564-1571,

https://doi.org/10.1021/jacs.2c08273.

[23]	Q. Jiang, P. Xiong, J. Liu, et al., “A Redox-Active 2D Metal-Organic Framework for Efficient Lithium Storage with Extraordinary High Capacity,” *Angew. Chem. Int. Ed.* 59, no. 13 (2020): 5273-5277,

https://doi.org/10.1002/anie.201914395.

[24]	K. Li, J. Yu, Z. Si, B. Gao, H.-g. Wang, Y. Wang, “One-dimensional π-d conjugated coordination polymer with double redox-active centers for all-organic symmetric lithium-ion batteries,” *Chem. Eng. J.* 450 (2022): 138052,

https://doi.org/10.1016/j.cej.2022.138052.

[25]	X. Sun, X. Yan, K. Song, et al., “A Pyrazine‐Based 2D Conductive Metal‐Organic Framework for Efficient Lithium Storage,” *Chin. J. Chem.* 41, no. 14 (2023): 1691-1696,

https://doi.org/10.1002/cjoc.202200819.

[26]	K. Wada, K. Sakaushi, S. Sasaki, H. Nishihara, “Multielectron-Transfer-based Rechargeable Energy Storage of Two-Dimensional Coordination Frameworks with Non-Innocent Ligands,” *Angew. Chem. Int. Ed.* 57, no. 29 (2018): 8886-8890,

https://doi.org/10.1002/anie.201802521.

[27]	K. Wada, H. Maeda, T. Tsuji, K. Sakaushi, S. Sasaki, H. Nishihara, “Tailoring the Electrochemical Properties of Two-Dimensional Bis(diimino)metal Coordination Frameworks by Introducing Co/Ni Heterometallic Structures,” *Inorg. Chem.* 59, no.15 (2020): 10604-10610,

https://doi.org/10.1021/acs.inorgchem.0c01055.

[28]	Z. Zhang, H. Yoshikawa, K. Awaga, “Monitoring the solid-state electrochemistry of Cu(2,7-AQDC) (AQDC = anthraquinone dicarboxylate) in a lithium battery: coexistence of metal and ligand redox activities in a metal-organic framework,” *J. Am. Chem. Soc.* 136, no. 46 (2014): 16112-16115,

https://doi.org/10.1021/ja508197w.

[29]	Z. Wang, G. Wang, H. Qi, et al., “Ultrathin two-dimensional conjugated metal-organic framework single-crystalline nanosheets enabled by surfactant-assisted synthesis,” *Chem. Sci.* 11, no. 29 (2020): 7665-7671,

https://doi.org/10.1039/d0sc01408g.

[30]	S. Gu, Z. Bai, S. Majumder, B. Huang, G. Chen, “Conductive metal–organic framework with redox metal center as cathode for high rate performance lithium ion battery,” *J. Power Sources* 429 (2019): 22-29,

https://doi.org/10.1016/j.jpowsour.2019.04.087.

[31]	P. Apostol, X. Lin, S. M. Dubois, et al., “A Single-Phase Mixed Ion-Electron Conducting Metal-Organic Framework,” *J. Am. Chem. Soc.* 148, no. 4 (2026): 4339,

https://doi.org/10.1021/jacs.5c18105.

[32]	Z. Wu, D. Adekoya, X. Huang, et al., “Highly Conductive Two-Dimensional Metal-Organic Frameworks for Resilient Lithium Storage with Superb Rate Capability,” *ACS Nano* 14, no. 9 (2020): 12016-12026

https://doi.org/10.1021/acsnano.0c05200.

[33]	C. Li, C. Zhang, J. Xie, et al., “Ferrocene-based metal-organic framework as a promising cathode in lithium-ion battery,” *Chem. Eng. J.* 404 (2021): 126463,

https://doi.org/10.1016/j.cej.2020.126463.

[34]	H. Dong, H. Gao, J. Geng, et al., “Quinone-Based Conducting Three-Dimensional Metal–Organic Framework as a Cathode Material for Lithium-Ion Batteries,” *J. Phys. Chem. C* 125, no. 38 (2021): 20814,

https://doi.org/10.1021/acs.jpcc.1c06870.

[35]	T. Cai, Z. Hu, Y. Gao, G. Li, Z. Song, “A Rationally Designed Iron–Dihydroxybenzoquinone Metal–Organic Framework as Practical Cathode Material for Rechargeable Batteries,” *Energy Storage Mater.* 50 (2022): 426-434,

https://doi.org/10.1016/j.ensm.2022.05.040.

[36]	K. Wakamatsu, S. Furuno, Y. Yamaguchi, et al., “Electron Storage Performance of Metal–Organic Frameworks Based on Tetrathiafulvalene–Tetrabenzoate as Cathode Active Materials in Lithium- and Sodium-Ion Batteries,” *ACS Appl. Energy Mater.* 6, no. 18 (2023): 9124,

https://doi.org/10.1021/acsaem.2c03537.

[37]	Z. Peng, X. Yi, Z. Liu, J. Shang, D. Wang, “Triphenylamine-Based Metal-Organic Frameworks as Cathode Materials in Lithium-Ion Batteries with Coexistence of Redox Active Sites, High Working Voltage, and High Rate Stability,” *ACS Appl. Mater. Inter.* 8, no. 23 (2016): 14578.

https://doi.org/10.1021/acsami.6b03418.

[38]	G. Ferey, F. Millange, M. Morcrette, et al., “Mixed-valence li/fe-based metal-organic frameworks with both reversible redox and sorption properties,” *Angew. Chem. Int. Ed.* 46, no. 18 (2007): 3259,

https://doi.org/10.1002/anie.200605163.

[39]	L. Hong, L. Li, Y. K. Chen-Wiegart, et al., “Two-dimensional lithium diffusion behavior and probable hybrid phase transformation kinetics in olivine lithium iron phosphate,” *Nat. Commun.* 8, no. 1 (2017): 1194,

https://doi.org/10.1038/s41467-017-01315-8.

[40]	C. Hong, Q. Leng, J. Zhu, et al., “Revealing the correlation between structural evolution and Li+diffusion kinetics of nickel-rich cathode materials in Li-ion batteries,” *J. Mater. Chem.* A 8, no. 17 (2020): 8540,

https://doi.org/10.1039/d0ta00555j.

[41]	Q. Liu, X. Su, D. Lei, et al., “Approaching the capacity limit of lithium cobalt oxide in lithium ion batteries via lanthanum and aluminium doping,” *Nat. Energy* 3, no. 11 (2018): 936,

https://doi.org/10.1038/s41560-018-0180-6.

[42]	B. N. P. Ping Yu, J. A. Ritter, and R. E. White, “Determination of the Lithium Ion Diffusion Coefficient in Graphite,” *J. Electrochem. Soc*. 146, no. 1 (1999): 8,

https://doi.org/10.1149/1.1391556.
